# Betting on the House: The Impact of Gambling on Homeownership in Australia

**DOI:** 10.1007/s10899-023-10217-y

**Published:** 2023-06-07

**Authors:** Kingsley Tetteh Baako, Kwabena Mintah, Sefa Awaworyi Churchill, Lisa Farrell

**Affiliations:** 1 School of Property, Construction and Project Management, RMIT University, Melbourne, VIC Australia; 2 School of Economics, Finance and Marketing, RMIT University, Melbourne, VIC Australia

**Keywords:** Gambling, Housing, Financial stress, Social capital, Homeownership, Australia

## Abstract

Gambling is commonly associated with social and economic disadvantage. In this paper we examine the impact of gambling on homeownership, using Australian panel data. We find that gambling is associated with a lower probability of homeownership. Specifically, our endogeneity corrected estimates show that an increase in problem gambling is associated with between 1.6 and 1.8 percentage point decrease in the probability of owning a home depending on the model. Our result show that financial stress and social capital are channels through which gambling influences the probability of homeownership.

## Introduction and Background

Gambling, defined as “the placement of a wager or bet on the outcome of a future uncertain event” (QGSO (Queensland Government Statistician’s Office), 2021) is commonplace in today’s society, with current (2021) estimates of the global gambling market standing at approximately US$ 516 billion with a compound annual growth rate of 10.8% (Research & Markets, 2021). This prevalence and growth in gambling around the world has spurred academic interest, yielding several studies into various forms of gambling. Studies have focused on understanding the psychology of gambling (Griffiths, 1990; James et al., 2017; Rickwood et al., 2010; Walker, 1992), gambling disorder and problem gambling (Awaworyi Churchill & Farrell, 2020a, 2020c; Brooks & Clark, 2019; Ioannidis et al., 2019; Petry et al., 2017; Potenza et al., 2019), the social, health and economic impacts of gambling (Awaworyi Churchill & Farrell, 2018, 2020b; Farrell & Fry, 2021; Latvala et al., 2019; Williams et al., 2021). This evidence suggests a range of social and economic harms are associated with gambling. Here we extend this literature by investigate the causal relationship between gambling and homeownership.

There is a consensus in the literature as to the benefits of homeownership. Traditionally, homeownership is seen to serve at least three human needs: a utility for consumption (Ioannides & Rosenthal, 1994), an investment asset (Ackerman et al., 2012), as well as a benefit to society (Rohe et al., 2013). It has positive effects on individuals as well as societies and economies. For instance, homeownership has been linked with positive influence on: wealth accumulation and social status (Yun & Evangelou, 2016), residential stability (Aaronson, 2000), higher civic participation (DiPasquale & Glaeser, 1999; Rotolo et al., 2010), higher educational and financial attainment and quality of life for homeowners’ children (Boehm & Schlottmann, 1999; Green & White, 1997; Green et al., 2012; Haurin et al., 2002), engaged parenting (Grinstein-Weiss et al., 2010), higher social capital (Manturuk et al., 2010), improved health care outcomes (Finnigan, 2014; Galster, 1987; Hamoudi & Dowd, 2013; Manturuk, 2012), lower incidence of neighborhood crimes (Kubrin, 2003; Lindblad et al., 2013), lower reliance on public assistance (Page-Adams & Vosler, 1997), among others.

Given these benefits, public policy makers in many countries encourage homeownership through policies that facilitate transitioning from renting to ownership. The factors that influence homeownership have thus been of significant interest. Studies have found that, at the micro level, homeownership is influenced by household income, number of children, marital status, household size, level of education, political affiliation, and a range of other socio-economic factors (see, e.g., Arimah, 1997; Constant et al., 2009; Garcia & Figueira, 2020; Nwuba et al., 2015). We extend existing knowledge on the factors that influence homeownership by investigating the impact of gambling on homeownership.

In terms of housing and gambling, existing studies have examined the relationship between housing and gambling, focusing mainly on housing (in)stability and problem gambling (Gattis & Cunningham-Williams, 2011; Guilcher et al., 2016), gambling and housing conditions (Stevens & Bailie, 2012) and gambling and homelessness (Antonetti & Horn, 2001; Castellani et al., 1996; Holdsworth & Tiyce, 2012; Landon et al., 2021). While there is conjecture and media reportage on the impact of gambling on homeownership (Ross, 2019; Whitten, 2021), there is little empirical evidence supporting the relationship.

There are several reasons why we expect gambling to influence the probability of homeownership. First, we expect gambling to impact homeownership through household savings. It has been established that gambling leads to financial harm (Browne et al., 2016), as gambling loss is inversely correlated with savings. With lower savings, a potential homeowner will struggle to save up the deposit to qualify for a mortgage, or the lumpsum to purchase the home outright, reducing the probability of homeownership. Through other gambling-related harm, such as health degradation (Antonetti & Horn, 2001; Blaszczynski et al., 1999), which can lead to higher healthcare costs, gambling indirectly leads to lower savings towards a home acquisition, further lowering homeownership prospects.

Similarly, gambling impacts the probability of homeownership through the financial stress it engenders. There is literary evidence to suggest that gambling can help increase or reduce financial stress (Koomson et al., 2022). In the former, gambling has been found to increase financial stress through financial loss and increased accrued debt (Mathews & Volberg, 2013). This financial stress then leads to the implementation of multiple coping mechanisms including working longer to garner funds to gamble or make up for funds lost to gambling, loss of employment due to gambling-induced psychological stress, and residence downsizing (Mathews & Volberg, 2013) (McComb et al., 2009). These impacts cumulatively affect the level of savings that a prospective homeowner can accumulate towards homeownership. In the end, gambling-induced financial stress can potentially lower the probability of homeownership for gamblers.

In the latter, a gambling win provides access to windfall cash gains which reduces financial stress and has been found to be directed towards purchasing homes and servicing mortgages, among other things (Furaker & Hedenus, 2009).

Gambling is also likely to impact homeownership prospects indirectly through social capital. Evidence suggests that gambling, especially problem gambling, leads to relationship breakdown and conflict (Browne et al., 2016; Dowling, 2014; Goodwin et al., 2017; Hing et al., 2020; Wardle et al., 2018). This breakdown in relationships attributed to gambling means that gamblers and their concerned significant others (Goodwin et al., 2017; Hodgins et al., 2007) are not able to maximize social cohesion, which is known to increase the prospects of homeownership (Brisson & Usher, 2007). In essence, social capital is a significant channel to influence the homeownership prospects of a household as it often provides, among other things, support in many forms which strengthen the prospects of a household in transitioning to homeownership. As (problem) gambling results in relationship breakdowns, and thus weakens social capital, a potential homeowner will lose the support that is often needed to transition from renting to homeownership.

Our focus on Australia is critical for at least two reasons. First, homeownership, although central to the “Great Australian dream” has been on the decline for Australians in recent years. Recent evidence indicates that Australian house price growth has outstripped income growth, leading to lowering homeownership rates, with some estimates yielding a 20% decline in homeownership rates in the past 3 decades to 2020 (Ong et al., 2015). This decline persists despite the ongoing COVID-19 pandemic which was expected to lower prices (Heath, 2020), and thus increase homeownership rates. Secondly, Australia is notorious for gambling. With a significant total gambling turnover of approximately $226 billion dollars, Australia has the highest gambling losses per capita (approximately AU$ 1300 per adult as of 2016/2017), with Australians losing nearly AUD $25 billion on legal forms of gambling in 2018/2019, and rising (Letts, 2018; QGSO (Queensland Government Statistician’s Office), 2021). With about 35% of adults spending money gambling each month., Australia is heralded as the nation that bets most in the world. Gambling has thus become a major policy issue in Australia and has yielded significant interest (AIHW, 2021), having been labelled an epidemic (Farrell & Fry, 2021). Given these circumstances, understanding the influence gambling has on homeownership will lend guidance to policy efforts to curb the rise of gambling as well as increase homeownership rates.

To empirically study the impact of gambling on homeownership, we employ two waves of panel data from the Household, Income and Labour Dynamics in Australia (HILDA) dataset. We find an increase in problem gambling is associated with a decline in the probability of homeownership and that financial stress and social capital are channels through which problem gambling influences homeownership. We also find that the financial stress is a more important mediator given that the mediating effect of financial stress is stronger than that of social capital.

This paper extends existing knowledge in at least five ways. First, it is the first to examine the direct and indirect relationship between gambling and homeownership. Existing studies look at other aspects of housing, without specifically considering the impact on homeownership. Essentially, we contribute to the wider group of studies that investigate the determinants of homeownership. Second, we contribute to the strand of literature that examines the economics and societal harms of gambling. While many studies examine the impacts of gambling, they are often narrative and theoretical. This study contributes empirical evidence to the knowledge on gambling. Third, given the policy significance of both gambling and homeownership, this study will provide evidence that will influence strategies and actions in both sectors, potentially strengthening efforts that will enhance the lives of many Australians (Farrell & Fry, 2021). Then, our examination of social capital as a transmission channel of gambling adds to the literature that focuses on the relationship between social capital and homeownership. Finally, examining financial stress as a mediator between gambling and homeownership extends the literature that investigates the consequences of financial stress.

The rest of the paper is structured as follows. “Data and variables” section is used to present the data and variables. In “Method” section, we discuss the empirical methodology. The results are presented and discussed in “Results” section, and the paper is concluded in “Conclusion” section.

## Data and Variables

We use data from the HILDA survey, which is a nationally representative panel survey for Australians. The survey, which is modelled after the United States Panel Study of Income Dynamics and the British Household Panel Survey, commenced in 2001 and has so far produced 20 waves of longitudinal data. Discussed in more detail in Watson and Wooden (2012), the survey collects information on the demographic, economic and social characteristics of households and individuals.

The gambling module in the HILDA survey are executed in waves 15 and 18, where respondents are asked a set of questions relating to household participation in gambling activities. Thus, our analysis is based on only waves 15 and 18 of the HILDA survey. Despite only having two waves of data, this is high quality population based longitudinal study with a rich information set for our purposes. There is a paucity of population based longitudinal gambling data and this is the only nationally representative large scale Australian dataset from which our research question can be addressed, to our knowledge.

### Gambling

Our main measure of gambling is the problem gambling severity index (PGSI) (Ferris & Wynne, 2001), which is a validated and widely used instrument to measure gambling severity, specially designed for inclusion in population based surveys i.e. sub-clinical samples (see, e.g., Awaworyi Churchill & Farrell, 2018, 2020c; Holtgraves, 2008; Korman et al., 2008; Orford et al., 2010; Trinh et al., 2022). We measure the PGSI using scores derived from a nine-item instrument that collects information on gambling participation and the consequences of problem gambling in the past 12 months. The nine items are based on the HILDA survey question that asks respondents: “Now thinking about the last 12 months: (1) Have you bet more than you could really afford to lose?; (2) Have you needed to gamble with larger amounts of money to get the same feeling of excitement?; (3) When you gambled, did you go back another day to try and win back the money you lost?; (4) Have you borrowed money or sold anything to get money to gamble?; (5) Have you felt that you might have a problem with gambling?; (6) Has gambling caused you any health problems, including stress or anxiety?; (7) Have people criticized your betting or told you that you had a gambling problem, regardless of whether or not you thought it was true?; (8) Has your gambling caused any financial problems for you or your household?; (9) Have you felt guilty about the way you gamble or what happens when you gamble?” Responses are on a four-point Likert scale, where 0 denotes ‘never’ and 3 denotes ‘almost always’. The PGSI scores range between 0 and 27, with higher scores representing greater gambling severity, is derived as the sum of responses across the instrument items.

In addition to the PGSI, we use alternative indicators of gambling for robustness. First, we derive a measure of gambling risk of harm based on the PGSI scores. Following the existing literature, we assign four risk (of harm) categories, namely non-problem gamblers, low-risk gamblers, moderate-risk gamblers and problem gamblers (Awaworyi Churchill & Farrell, 2020b; Koomson et al., 2022). Non-problem gamblers have a PGSI score of 0 and are respondents who did not engage in problematic gambling behaviour. Low-risk gamblers are those with PGSI scores of 1 or 2, while moderate-risk gamblers have scores of 3 to 7. Problem gamblers are respondents with PGSI scores of 8 to 27. Using the foregoing classification based on the PGSI scores, we derive an ordinal scale of gambling risk status where 1, 2, 3 and 4 represents non-problem, low-risk, moderate-risk and problem gamblers, respectively. Thus, a movement up the harm-risk status scale represents higher risk of gambling related harms. Second, we derive four binary variables of gambling behaviour capturing each gambling risk category. Thus, in alternative regressions, we use binary variables capturing the respondents in each gambling risk category. Third, we also consider the average expenditure on gambling as a share of income. The HILDA survey provides information on average monthly expenditure on gambling. We derive an annual expenditure from this information and deflate with annual income. Fourth, the HILDA survey asks participants about gambling activities they engage in. We generate binary variables for each gambling activity respondents engage in. The activities captured include gambling using scratch cards, lotto or lottery games, bingo, keno, private betting, poker, casino table games, poker machines, horse and dogs race betting and sports betting.

### Homeownership

To measure homeownership, we draw on information from the HILDA survey question: “Do you (or any other members of this household) own this home, rent it, or do you live here rent free?” Consistent with the existing literature, our measure of homeownership is a binary variable set equals to one if the respondent answered that either they, or another member of their household, owned the home and zero if otherwise (Awaworyi Churchill et al., 2022; Borjas, 2002; Constant et al., 2009; Mintah et al., 2022; Munyanyi et al., 2021). Given the alternative options (rent it or live here for free) it can reasonably be assumed that homeownership is defined, in the standard way as owning outright or owned via a mortgage. Our data does not allow us to distinguish between these two groups. It should also be noted that the question applies to the place of residence at the time of the interview and so we do not capture ownership of second homes (holiday homes) or investment properties via this measure.

### Financial Stress and Social Capital

To better understand the causal pathway between gambling and homeownership we consider financial stress and social capital as mediators. To measure financial stress, we draw on information from the HILDA survey on respondents’ experience of economic hardship and financial stress. The HILDA survey asks respondents to indicate with indicate with a “Yes” or “No” if since the beginning of the reference year (i.e., year survey was taken) they experience any of seven issues pertaining to financial or economic hardship. Specifically, the survey asks: “Did any of the following happen to you because of a shortage of money?: (1) could not pay electricity, gas or telephone bills on time; (2) could not pay the mortgage or rent on time; (3) pawned or sold something; (4) went without meals; (5) was unable to heat home; (6) asked for financial help from friends or family; and (7) asked for help from welfare/community organisations”. Consistent with the existing literature, we sum up the responses to all seven questions to generate an indicators of financial stress with scores ranging from 0 to 7 (Breunig et al., 2019; Koomson et al., 2022).

To measure social capital, we focus on the dimension of support (Awaworyi Churchill & Farrell, 2020c; Awaworyi Churchill et al., 2023; Hewitt et al., 2010; Milner et al., 2016), and construct and index based on a 10-item instrument from the HILDA survey, which asks respondents about their sentiments regarding the support they are likely to receive from friends and families. The HILDA survey asks: “The following statements have been used by many people to describe how much support they get from other people. How much do you agree or disagree with each?: (1) I enjoy the time I spend with the people that are important to me; (2) I seem to have a lot of friends; (3) There is someone who can always cheer me up when I am down; (4) When I need someone to help me out, I can usually find someone; (5) When something's on my mind, just talking with the people I know can make me feel better; (6) People don't come and visit as much as I would like; (7) I often need help from other people but can't get it; (8) I don't have anyone that I can confide in; (9) I have no one to lean on in times of trouble; (10) I often feel very lonely”. The responses are coded on 1–7 Likert scale, where 1 represents ‘strongly disagree’ and 7 represents ‘strongly agree’. We reverse code responses to questions 6 to 10 and derive our index of social capital as the average of responses to the 10 items with higher scores indicating stronger social capital.

### Covariates

We control for demographic and socioeconomic factor correlated with homeownership as this is our dependent variable. Consistent with the literature, we control for gender, age, marital status, employment status, and education status, of the household reference person. We also control for household size, household income, and geographic location. Table 7 presents a summary and description of variables.

## Method

Our estimates are based on the following model:

1 $$H_{it} = \alpha + \beta_{1} G_{it} + \mathop \sum \limits_{n} \beta_{n} X_{n,it} + \alpha_{s} + \mu_{t} + \varepsilon_{it}$$

where $H_{it}$ is homeownership in time $t$ capturing the HILDA wave; $G$ is our measure of behaviour; $X$ is a set of factors correlated with the probability of homeownership; $\alpha_{s}$ represents state fixed effects; $\mu_{t}$ represents time fixed effects, and $\varepsilon$ is the error term. For ease of interpretation, we estimate Eq. (1) as a linear probability model although we find that a logit model yielded similar results in regard to the direction of effects.

To address endogeneity which may arise due to measurement error or omitted variable bias, we adopt an instrumental variable (IV) model where, consistent with the literature, we instrument for gambling behaviour using the number of electronic gaming machines in each state (Farrell & Fry, 2021). The number of gambling machines in an area is typically considered as a proxy for the opportunities available to gamble and is thus correlated with gambling behaviour. This instrument further satisfies the exclusion restriction given that the number of gambling or gaming machines in an area should influence the probability of homeownership only via gambling behaviour. To ensure that our results are robust, we also complement the traditional two stage least squares (2SLS) approach with the Lewbel (2012) 2SLS approach which does not rely on a valid exclusion restriction but constructs internal instruments using heteroskedastic covariance restrictions. This approach is widely used in the literature in the absence of traditional external instruments or as robustness checks when external instruments are available (see, e.g., Ambrey & Fleming, 2014; Awaworyi Churchill & Smyth, 2019; Munyanyi et al., 2020; Prakash et al., 2020, 2022).

## Results

Columns 1 and 2 of Table 1 report the results for the association between PGSI and homeownership, while Columns 3 and 4 report results for the association between the risk status ordinal scale and homeownership.^1^ Columns 1 and 3 present results from a parsimonious model that excludes relevant covariates, while results in Columns 2 and 4 include all covariates. Consistently, we find that an increase in gambling severity is associated with a decline in the probability of homeownership. Specifically, focusing the complete model for the PGSI scores, we find that a unit increase in the PGSI score is associated with a 1.1 percentage point decrease in the probability of owning a home. Turning to the results for risk status in Column 4, we find that a shift from one risk category to a higher risk category is associated with a 3.9 percentage point decrease in the probability of owning a home.

**Table 1:** Gambling and homeownership (baseline)

	Dependent variable: Homeownership			
	PGSI	PGSI	Risk Status	Risk Status
	(1)	(2)	(3)	(4)
Gambling	− 0.023***	− 0.011***	− 0.079***	− 0.039***
	(0.002)	(0.002)	(0.008)	(0.007)
State fixed effect	Yes	Yes	Yes	Yes
Time fixed effect	Yes	Yes	Yes	Yes
Controls	No	Yes	No	Yes
Observations	15,749	15,717	15,749	15,717
R-squared	0.010	0.289	0.011	0.289

The outcome variable for each column is the homeownership binary variable

The measure of gambling in columns (1) and (2) is the PGSI, while the measure in Columns (3) and (4) is the risk status ordinal scale

Cluster robust standard errors in parentheses

***p < 0.01, **p < 0.05, *p < 0.1

**Table 2:** IV results

	Dependent variable: Homeownership					
	PGSI	Risk Status	PGSI	Risk Status	PGSI	Risk Status
	(1)	(2)	(3)	(4)	(5)	(6)
Gambling	− 0.016***	− 0.054***	− 0.018***	− 0.040***	− 0.017***	− 0.043***
	(0.003)	(0.012)	(0.003)	(0.013)	(0.003)	(0.010)
State fixed effect	Yes	Yes	Yes	Yes	Yes	Yes
Time fixed effect	Yes	Yes	Yes	Yes	Yes	Yes
Controls	Yes	Yes	Yes	Yes	Yes	Yes
Observations	15,717	15,717	15,717	15,717	15,717	15,717
*First stage*						
Gambling machines	0.363***	0.153***			0.112***	0.547***
	(0.014)	(0.039)			(0.013)	(0.012)
F-statistics	247.95	338.06	156.62	135.17	110.39	209.33
J *p*-value	–	–	0.1948	0.2620	0.0646	0.2651

See Table 1 notes

Robust standard errors in parentheses

***p < 0.01, **p < 0.05, *p < 0.1

**Table 3:** Gambling risk categories

	Dependent variable: Homeownership			
	Non-gambler	Low-risk gambler	Medium-risk gambler	Problem gambler
	(1)	(2)	(3)	(4)
*Panel A*				
Gambling	0.071***	− 0.071***	− 0.093***	− 0.154***
	(0.013)	(0.017)	(0.015)	(0.031)
Observations	15,717	15,717	15,717	15,717
R-squared	0.287	0.286	0.285	0.287
*Panel B*				
Gambling	n.a	− 0.074***	− 0.081***	− 0.159***
	n.a	(0.017)	(0.019)	(0.031)
Observations	n.a	15,109	14,829	14,584
R-squared	n.a	0.286	0.287	0.292

See Table 1 notes

Robust standard errors in parentheses

***p < 0.01, **p < 0.05, *p < 0.1

Of the control variables included in the models, we find that age is positively associated with homeownership. Being female, being divorced or separated is associated with a lower probability of homeownership. Being employed, having a bigger household size, being better educated, having higher income, being married, in a de facto relationship or widowed (relative to being single) increase the probability of homeownership. Long-term illness and living in a metropolitan area reduce the probability of homeownership.

Table 2 reports 2SLS results for the relationship between gambling and homeownership. Columns 1 and 2 report 2SLS results using number of gaming machines as instruments. Columns 3 and 4 report results using the Lewbel (2012) 2SLS approach with internally generated instruments only, while Columns 5 and 6 report results from the Lewbel (2SLS) estimations that rely on both external and internal instruments. Across all columns, the first stage F statistics are greater than 10, suggesting that at the 5 per cent significance level, our instruments are not weakly correlated with gambling behaviour. The positive sign on number of gaming machines is also consistent with expectations and previous literature (Farrell & Fry, 2021). For regressions with multiple instruments, we do not reject the null hypothesis for the overidentifying restriction test. Across all columns, we find that the 2SLS results confirm the negative relationship between gambling severity and homeownership. However, the 2SLS estimates are higher than the baseline estimates, suggesting that endogeneity generates a downward bias in our baseline estimates. Specifically, we find that, depending on the model, a unit increase in the PGSI score is associated with between 1.6 and 1.8 percentage point decrease in the probability of owning a home, while we find between 4.0 and 5.4 percentage point decrease in the probability of owning a home for risk status.

In Table 3, we conduct two set of analyses that examine the effects of the different harm-risk categories based on the PGSI scores. First, in Panel A, we use binary variables to capture each of the risk categories (i.e., non-problem gamblers, low-risk gamblers, moderate risk gamblers and problem gamblers) and examine the effects on homeownership. Second, in Panel B, we focus on a similar construct where for each binary variable, the reference category is strictly respondents who are non-problem gamblers. In Panel A, we find that being a non-problem gambler is associated with a higher probability of owning a home, while in Panels A and B, being a low-risk, medium-risk or problem gambler is associated with a lower probability of homeownership. Additionally, the results suggest that the probability of homeownership decline is higher for problem gamblers compared to medium-risk gamblers, and higher for medium risk gamblers compared to low-risk gamblers. In Table 10, we further consider an analysis where we include low-risk gamblers, moderate risk gamblers and problem gamblers in one model (leaving out non-problem gamblers as base). We find that the results are robust.

In Table 4, we examine the impact of gambling expenditure and engagement in different gambling activities on the probability of homeownership. We find that an increase in the share of income spend on gambling is associated with a decline in the probability of homeownership. Regarding the gambling activities, we find that engaging in Bingo, lottery games, casino table games and sports betting are associated with a decline in the probability of homeownership, while the coefficients on the other gambling activities are statistically insignificant.

**Table 4:** Gambling expenditure and activities

	Dependent variable: Homeownership									
	Expenditure	Scratch	Bingo	Lotto	Keno	Bet	Casino	Poker	Race	Sports
	(1)	(2)	(3)	(4)	(5)	(6)	(7)	(8)	(9)	(10)
Gambling	− 0.006***	− 0.009	− 0.114***	− 0.022***	− 0.006	− 0.018	− 0.064**	0.010	0.015	− 0.031*
	(0.001)	(0.012)	(0.033)	(0.007)	(0.018)	(0.028)	(0.029)	(0.031)	(0.013)	(0.017)
Observations	15,688	15,688	15,688	15,688	15,688	15,688	15,688	15,688	15,688	15,688
R-squared	0.286	0.287	0.288	0.288	0.287	0.288	0.288	0.287	0.288	0.288

Robust standard errors in parentheses

***p < 0.01, **p < 0.05, *p < 0.1

### Potential Channel Analysis

In what follows, we examine if financial stress and social capital are channels through which gambling influences the probability of homeownership. For financial stress and social capital to qualify as channels of influence, in addition to being correlated with gambling, they should also be correlated with homeownership and their inclusion as additional covariates in the regression linking gambling to homeownership should reduce the magnitude of the coefficient on gambling (see, e.g., Alesina & Zhuravskaya, 2011).

As a first step, Table 5 reports results for the effects of gambling on financial stress and social capital. Columns 1 report results for effects on financial stress, while Column 2 reports effects on social capital. We find that an increase in problem gambling is associated with higher financial stress and lower social capital. Next, in Table 6 we proceed to include financial stress and social capital as additional covariates in alternative models linking gambling to homeownership. Given that the inclusion of the mediators reduces the number of observations, in Column 1 we rerun the regression linking gambling with homeownership to ensure that the same sample is used in the potential channel analysis when we relate financial stress and social capital to homeownership. From Column 2, we find that an increase in financial stress is associated with a decline in the probability of homeownership, while in Column 3, an increase in social capital is associated with an increase in the probability of homeownership. Further, compared with the coefficient on gambling in Column 1, we find that the inclusion of financial stress and social capital as additional covariates reduces the coefficient on gambling, although only marginally for social capital. This confirms that financial stress and social capital are potential channels that link gambling to homeownership although financial stress is a more important mediator given that the mediating effect of financial stress is stronger than that of social capital.

**Table 5:** Impact of gambling on mediators

	Financial Stress	Social capital
	(1)	(2)
Gambling (PGSI)	0.018***	− 0.058***
	(0.002)	(0.009)
State fixed effect	Yes	Yes
Time fixed effect	Yes	Yes
Controls	Yes	Yes
Observations	16,282	16,282

Robust standard errors in parentheses

***p < 0.01, **p < 0.05, *p < 0.1

**Table 6:** Impact of mediators on homeownership

	Dependent variable: Homeownership		
	(1)	(2)	(3)
Gambling (PGSI)	− 0.023***	− 0.014***	− 0.021***
	(0.002)	(0.002)	(0.002)
Financial Stress		− 0.369***	
		(0.011)	
Social capital			0.018***
			(0.001)
Observations	15,749	15,749	15,749
R-squared	0.010	0.077	0.038

Robust standard errors in parentheses

***p < 0.01, **p < 0.05, *p < 0.1

To ensure that our results are robust, we consider the PROCESS mediation approach as alternative approach to mediation analysis (Preacher & Hayes, 2008). Following Wiklund et al. (2017), we use 1,000 replications of bootstrapping and the bias-corrected percentile approach to deal with potential non-normality in our data. The advantages of specifying and testing a single multiple mediation model, such as PROCESS, instead of separate simple mediation models include: (1) the ability to determine if the overall effect of mediation exists; (2) the ability to identify the extent to which each of the mediating variables intervenes between the independent and dependent variables in the presence of other potential mediators; (3) limiting missing parameter bias; and (4) the ability to determine relative magnitudes of specific indirect effects (Preacher & Hayes, 2008). The results reported in Table 11 reinforce the finding that financial stress and social capital are channels that link gambling to homeownership. Specifically, we find that a unit increase in the PGSI score is associated with a 2.3% increase in financial stress and 11.8% decline in social capital. Thus, by increasing financial stress and diminishing social capital, gambling reduces the probability of homeownership.

## Conclusion

While media and theoretical conjecture have been proffered the existence of a relationship between gambling and homeownership, there is scant evidence to support this. This study sought to empirically examine the relationship between gambling and homeownership, filling an important knowledge gap. Additionally, this study examines the potential of social capital and financial stress as mediators in the gambling-homeownership nexus. Employing the high-quality HILDA survey data, we investigated whether Australians are betting away their homeownership prospects.

Our overall finding is that problem gambling is associated with a decline in the probability of homeownership. This finding was consistent across different measures of gambling and robustness checks. For instance, using the PGSI, we found that a unit increase in the PGSI score is associated with a 1.1% decline in the probability of owning a home. The gambling risk status results also suggest that a shift from one risk category to a higher risk category is associated with an even higher decline in the probability of owning a home. Further, we found that social capital and financial stress are both channels through which gambling influences the probability of owning a home, although financial stress is a more important mediator.

To sum, Australians *are* betting on the house, the evidence suggests. The more Australians bet, the less likely they are to be able to own a home. With homeownership rates are at a record low of 67% (APH, 2022), increased efforts by the government to improve homeownership rates need to consider the impact of gambling. By directing efforts at reducing the severity of gambling, the government will indirectly be influencing the probability of Australians owning a home. Specifically, given the observation of a consistently lower probability of homeownership with a higher risk status, efforts can be directed at helping problem gamblers lower their risk status progressively over time. The mediator analysis shows that policy efforts directed at alleviating financial stress among gamblers will aid in lowering homeownership decline since we found financial stress to be a stronger channel of transmission of gambling on homeownership. Efforts aimed at promoting social capital can also be useful. One limitation of the current study is that while financial stress and gambling are likely to influence gambling behaviour, we focus on the role of these variables as mediators and thus, examine how gambling influences homeownership through these constructs. It is, however, likely that financial stress or lower social capital can lead to problem gambling, which then reduces homeownership. This lends support to the need for interventions that aim at reducing financial stress as well as promote social capital. Thus, not only is it relevant to target problem gambling but it also critical for interventions to examine the pathways through which gambling influences homeownership.

Based on the findings from this study, government efforts at curbing gambling to increase homeownership rates can be targeted specifically at participation in and access to Bingo, lottery games, casino table games and sports betting, as these forms of gambling have been found to be positively associated with a decline in homeownership probability.

Overall, this paper adds to the growing body of evidence of economic and societal related gambling harms experienced in Australia and acts as a warning for other countries experiencing growth in their betting and gaming sector. The findings also hold relevance for countries with similar socioeconomic and cultural patterns as Australia. However, we are not able to generalize the findings to other populations with very different characteristics.

## Appendix

See Tables

**Table 7:** Description and summary statistics

Variable	Description	Mean	SD
Homeownership	Dummy variable equals 1 if respondents is a homeowner	0.665	0.472
PGSI score	PGSI gambling scale (9-item scale with maximum score of 27)	0.325	1.619
Gambling risk	1–4 gambling risk scale based on PGSI scores	1.135	0.493
Non-problem gambler	Dummy variable equals 1 if respondent is a PGSI non-problem gambler (PGSI score 0)	0.916	0.278
Low-risk gambler	Dummy variable equals 1 if respondent is a PGSI low-risk gambler (PGSI score 1 or 2)	0.0450	0.207
Moderate risk gambler	Dummy variable equals 1 if respondent is a PGSI moderate-risk gambler (PGSI score 3 to 7)	0.0272	0.163
Problem gamblers	Dummy variable equals 1 if respondent is a PGSI problem gambler (PGSI score 8 or above)	0.0120	0.109
Age	Age of respondent	48.42	17.73
Male	Dummy variable equals 1 if respondent is male	0.591	0.492
Female	Dummy variable equals 1 if respondent is female	0.409	0.492
Employed	Dummy variable equals 1 if respondent is employed	0.674	0.469
Unemployed	Dummy variable equals 1 if respondent is unemployed	0.0258	0.159
Not in labour force	Dummy variable equals 1 if respondent is not in the labour force	0.300	0.458
Log(disposable Income)	Continuous variable for the log of household income	10.80	1.172
Married	Dummy variable equals 1 if respondent is married	0.449	0.497
Defacto	Dummy variable equals 1 if respondent is in a de facto relationship	0.128	0.334
Separated	Dummy variable equals 1 if respondent is separated	0.0445	0.206
Divorced	Dummy variable equals 1 if respondent is divorced	0.0987	0.298
Widowed	Dummy variable equals 1 if respondent is widowed	0.0798	0.271
Postgraduate	Dummy variable equals 1 if respondent’s highest education level achieved is masters or doctorate	0.0508	0.220
Graduate diploma	Dummy variable equals 1 if respondent’s highest education level achieved is graduate diploma	0.0587	0.235
Bachelor	Dummy variable equals 1 if respondent’s highest education level achieved is bachelor or honours	0.142	0.349
Diploma	Dummy variable equals 1 if respondent’s highest education level achieved is a diploma	0.0960	0.295
Certificate	Dummy variable equals 1 if respondent’s highest education level achieved is a certificate	0.237	0.425
Year12	Dummy variable equals 1 if respondent’s highest education level achieved is year 12 or below	0.127	0.332
Disability	Dummy variable equals 1 if respondent is disabled or has a long-term illness	0.259	0.438
household size	Continuous variable for the log of household size	0.741	0.561

^7^,

**Table 8:** Gambling and homeownership (Full results)

Variables	(1)	(2)
	Owner	Owner
Gambling	− 0.011***	− 0.039***
	(0.002)	(0.007)
Age	0.546***	0.547***
	(0.012)	(0.012)
Female	− 0.018***	− 0.019***
	(0.007)	(0.007)
Employed	0.084***	0.084***
	(0.009)	(0.009)
Income	0.039***	0.039***
	(0.005)	(0.005)
Married	0.182***	0.181***
	(0.013)	(0.013)
De facto	0.024*	0.024*
	(0.014)	(0.014)
Separated	− 0.062***	− 0.062***
	(0.021)	(0.021)
Divorced	− 0.036**	− 0.037**
	(0.016)	(0.016)
Widowed	0.134***	0.133***
	(0.016)	(0.016)
Postgrad	0.104***	0.103***
	(0.014)	(0.014)
Graduate diploma	0.142***	0.141***
	(0.013)	(0.013)
Bachelor	0.109***	0.108***
	(0.011)	(0.011)
Diploma	0.107***	0.107***
	(0.012)	(0.012)
Certificate	0.063***	0.063***
	(0.010)	(0.010)
year12	0.068***	0.068***
	(0.012)	(0.012)
Long-term illness	− 0.068***	− 0.069***
	(0.008)	(0.008)
Household size	0.045***	0.045***
	(0.009)	(0.009)
Metro area	− 0.049***	− 0.049***
	(0.007)	(0.007)
Constant	− 2.073***	− 2.033***
	(0.066)	(0.067)
Observations	15,717	15,717
R-squared	0.289	0.289

Robust standard errors in parentheses

****p* < 0.01, ***p* < 0.05, **p* < 0.1

^8^,

**Table 9:** Gambling and homeownership (Logit regressions)

	Dependent variable: Homeownership			
	PGSI	PGSI	Risk Status	Risk Status
	(1)	(2)	(3)	(4)
Gambling	− 0.100***	− 0.059***	− 0.331***	− 0.217***
	(0.012)	(0.013)	(0.033)	(0.039)
State fixed effect	Yes	Yes	Yes	Yes
Time fixed effect	Yes	Yes	Yes	Yes
Controls	No	Yes	No	Yes
Observations	15,749	15,717	15,749	15,717

Notes: The outcome variable for each column is the homeownership binary variable

The measure of gambling in columns (1) and (2) is the PGSI, while the measure in Columns (3) and (4) is the risk status ordinal scale

Cluster robust standard errors in parentheses

****p* < 0.01, ***p* < 0.05, **p* < 0.1

^9^,

**Table 10:** Gambling risk categories

	Dependent variable: Homeownership	
	(1)	(2)
Low-risk gambler	− 0.080***	− 0.075***
	(0.024)	(0.017)
Medium-risk gambler	− 0.105***	− 0.090***
	(0.019)	(0.019)
Problem gambler	− 0.324***	− 0.159***
	(0.035)	(0.031)
Controls	No	Yes
Observations	15,749	15,717
R-squared	0.012	0.288

The outcome variable for each column is the homeownership binary variable

Column (1) does not include covariates while Column (2) includes the full set of covariates

Cluster robust standard errors in parentheses

****p* < 0.01, ***p* < 0.05, **p* < 0.1

^10^ and

**Table 11:** Causal mediation approach (Liu et al., 2014)

Dependent variable: Self-employment	Indirect effect		Direct effect	
	Estimate	95% confidence interval	Estimate	95% confidence interval
PGSI=>Financial stress=>homeownership	0.023***	[0.0195, 0.0258]	− 0.020***	[− 0.0263, − 0.0145]
	(0.002)		(0.003)	
PGSI=>Social capital=>homeownership	− 0.118*	[− 0.2419, 0.0056]	− 0.021***	[− 0.0278, − 0.0149]
	(0.063)		(0.003)	

Bootstrap standard errors in parentheses

****p* < 0.01, ***p* < 0.05, **p* < 0.1

^11^.

## Footnotes

1 Full set of results including all covariates are reported in Appendix Table 8. Logit results are also reported in Table 9 and the direction of effects are consistent with our main results.
